# Development of the Mugla Score: an association-based tool for risk stratification in emergency department patients with rhabdomyolysis

**DOI:** 10.1007/s11739-025-04009-y

**Published:** 2025-06-11

**Authors:** Ömer Faruk Karakoyun, Fulden Cantaş Türkiş, Yalcin Golcuk, Mehmet Reha Yılmaz, Burcu Kaymak Golcuk

**Affiliations:** 1 Emergency Medicine Service, Muğla Training and Research Hospital, Muğla, Turkey; 2 Department of Biostatistics, Faculty of Medicine, Muğla Sıtkı Koçman University, Muğla, Turkey; 3 Department of Emergency Medicine, Faculty of Medicine, Muğla Sıtkı Koçman University, Muğla, Turkey; 4 Emergency Department, University Hospitals Coventry & Warwickshire, Coventry, UK; 5 Clinical Biochemistry Service, Muğla Training and Research Hospital, Muğla, Turkey

**Keywords:** Rhabdomyolysis, Acute kidney injury, Renal replacement therapy, Emergency department, Scoring system, Prognosis

## Abstract

Rhabdomyolysis is a potentially life-threatening syndrome characterized by skeletal muscle breakdown and systemic release of intracellular components, often resulting in acute kidney injury or death. Early risk stratification remains challenging in the emergency department (ED) setting due to heterogeneous presentations and unpredictable outcomes. To develop and internally validate the Mugla Score—a pragmatic, association-based tool for predicting adverse outcomes in ED patients with rhabdomyolysis. In this retrospective, single-center cohort study, adult ED patients with serum creatine kinase ≥ 1000 U/L between July 1, 2019, and July 1, 2024, were included. The primary outcome was a composite of renal replacement therapy or 90-day mortality. Multivariable logistic regression identified independent predictors, which were assigned weighted point values. Internal validity was assessed using five-fold cross-validation and 1,000-iteration bootstrap resampling. Among 1031 patients (mean age: 49.0 ± 21.8 years; 75.9% male), 109 (10.6%) experienced the composite outcome. Seven variables were independently associated with adverse events: age ≥ 50 years, platelet count ≤ 170 × 10^3^/μL, MCHC ≤ 32.8 g/dL, calcium ≤ 8.5 mg/dL, ALP ≥ 115 U/L, BEecf ≤  − 6 mmol/L, and etiological classification. The Mugla Score (range: 0–12.5) showed strong discrimination (AUC: 0.861, 95% CI: 0.824–0.898). A threshold of ≥ 4 points yielded a 97% negative predictive value. The Mugla Score provides a clinically interpretable, ED-focused tool for early risk stratification in rhabdomyolysis. While internally validated, external prospective studies are needed to assess generalizability prior to routine clinical adoption.

**Supplementary Information:**

The online version contains supplementary material available at 10.1007/s11739-025-04009-y.

## Introduction

Rhabdomyolysis is a complex clinical syndrome resulting from the breakdown of skeletal muscle and the subsequent release of intracellular contents—including myoglobin, potassium, and creatine kinase (CK)—into the systemic circulation. Clinical severity ranges from asymptomatic enzyme elevation to life-threatening complications such as electrolyte imbalance, acute kidney injury (AKI), and death [1, 2]. Global epidemiological data suggest that rhabdomyolysis contributes to 10–25% of AKI cases in hospitalized patients, with more than 200,000 cases annually in the United States alone. In emergency departments (EDs), rapid and accurate identification of patients at risk for severe outcomes remains a substantial challenge for clinicians [3].

Several prognostic scoring tools have been proposed to aid in the risk stratification of rhabdomyolysis patients, with the McMahon Score being the most widely cited. However, this score was not originally developed for use in ED populations and includes laboratory parameters—most notably serum phosphate—that are not routinely measured during the initial phase of ED care. Moreover, its applicability is restricted to patients with creatine kinase (CK) levels ≥ 5000 U/L, thereby excluding a substantial proportion of individuals presenting in the early or moderate stages of the disease [4]. Similarly, other models such as the Liu Score, which leverage machine learning techniques, have been proposed to address certain limitations [5]. Nonetheless, both the McMahon and Liu Scores were developed based on cohorts of critically ill patients in intensive care settings, limiting their generalizability to broader ED populations. Furthermore, these models do not account for the etiological heterogeneity of rhabdomyolysis, which may have a significant impact on patient prognosis [4, 5].

Given these limitations, there is a need for a simple, transparent, and clinically interpretable scoring system tailored for use in emergency care. An ideal model should rely solely on readily available laboratory and clinical data obtainable within the first hour of ED evaluation, be adaptable to the varied etiologies of rhabdomyolysis, and offer practical utility without requiring computational infrastructure.

In response to these gaps, we developed and internally validated the Mugla Score—a risk stratification tool based on routinely collected ED data, designed to identify factors associated with adverse outcomes in rhabdomyolysis.

## Methods

### Study design and setting

This retrospective, single-center, cohort study was conducted at the ED of a university-affiliated training and research hospital in [blinded for peer review], Turkey, from July 1, 2019, to July 1, 2024. The hospital, with a 50-bed ED and approximately 150,000 annual visits, offered an ideal setting for developing rhabdomyolysis assessment tools. Ethical approval was granted by the Institutional Review Board of [blinded for peer review] University (decision number: 240035/12). The study adhered to the principles outlined in the Declaration of Helsinki. Due to the retrospective nature of the analysis, the requirement for obtaining written informed consent from patients was waived. This manuscript adheres to the STROBE (Strengthening the Reporting of Observational Studies in Epidemiology) guideline for reporting observational cohort data and to the TRIPOD (Transparent Reporting of a Multivariable Prediction Model for Individual Prognosis or Diagnosis) guideline for the development and internal validation of the prediction model.

Assuming a medium effect size (odds ratio = 1.5), an adverse outcome prevalence of approximately 10%, a two-sided alpha level of 0.05, and a target power of 80%, the minimum required sample size for model development was estimated to be approximately 850 participants.

### Selection of participants

All adult patients (≥ 18 years) diagnosed with rhabdomyolysis, defined by serum CK ≥ 1000 IU/L at the time of ED admission, were included. Exclusion criteria were: (i) patients < 18 years, (ii) elevated CK due to acute coronary syndrome, stroke, or hemorrhage, (iii) rhabdomyolysis developed after admission, (iv) patients on chronic renal replacement therapy (RRT), (v) transferred patients, (vi) patients lost to follow-up within 3 months, or (vii) incomplete records for key variables.

To minimize selection bias, a comprehensive electronic screening of all ED admissions during the study period was performed using diagnostic and laboratory codes. A priori power analysis was not conducted due to the exhaustive inclusion of all eligible patients in the specified timeframe.

### Definitions and determination etiological factor groups

Rhabdomyolysis was defined as a serum CK level ≥ 1000 IU/L, reflecting clinically significant skeletal muscle injury. Renal replacement therapy (RRT) includes peritoneal dialysis, intermittent hemodialysis, and continuous renal replacement modalities. Adverse outcomes were defined as the initiation of RRT or all-cause mortality within 90 days of ED presentation. This composite endpoint was chosen to represent clinically meaningful deterioration necessitating escalation of care or resulting in death and is consistent with prior outcome definitions used in rhabdomyolysis and acute kidney injury research.

Rhabdomyolysis is a complex disorder with numerous potential etiologies, and currently, no universally accepted classification system exists. In our study, etiological factors were systematically categorized into five distinct classifications to enhance the clarity and precision of our analysis [1, 6]. Group 1 encompassed etiological factors such as alcohol consumption, endocrine abnormalities, metabolic derangements, myopathies, and infections, including COVID-19. Group 2 involved trauma-related causes, surgical procedures, seizure activity, and extreme physical exertion. Group 3 encompassed hyperosmolar conditions, specifically general medical states associated with dehydration, including prolonged exposure to elevated temperatures and insufficient fluid intake. Group 4 comprised rhabdomyolysis induced by pharmacologic agents, exposure to various venoms, including those from snakes and scorpions, as well as environmental toxins. Group 5 encompassed factors that did not fit within the predefined categories and were thus classified as “other.” Detailed definitions of etiological subgroups are provided in Online Appendix A.

### Data collection and laboratory timing

Potentially eligible cases were identified through a query of the hospital’s electronic medical records system at [blinded for peer review] Training and Research Hospital. Data were abstracted using a standardized electronic spreadsheet to ensure consistent and accurate capture of demographic information, etiological classifications, and baseline clinical variables.

Laboratory parameters—including complete blood count, serum biochemistry, and venous blood gas results—were obtained within the first hour of ED triage and prior to any therapeutic intervention. These values, acquired as part of routine care, reflect the patient’s acute physiological status at presentation. Since the interval between symptom onset and ED arrival was inconsistently documented, laboratory timing was standardized using triage timestamps.

Secondary outcomes such as hospital admission status and length of stay (LOS) were recorded. The primary composite outcome—RRT or all-cause mortality within 90 days—was verified using institutional medical records.

Anonymized data were stored on password-protected institutional servers, accessible only to authorized study personnel, in compliance with institutional data governance policies.

### Statistical analysis

Normality of continuous variables, including age and all laboratory parameters, was assessed using the Kolmogorov–Smirnov test. Since these variables exhibited non-normal distributions—with skewness and/or outliers—the Mann–Whitney *U* test was used as a non-parametric alternative for between-group comparisons. Continuous variables were summarized as mean ± standard deviation (SD) or median (min–max), and categorical variables as frequencies and percentages. Categorical variables were expressed as frequencies and percentages and compared using the chi-square test.

Missing data were evaluated using Little’s missing completely at random (MCAR) test, which indicated that data were MCAR. As the proportion of missing data was below 5% for each variable and the MCAR assumption held, a complete case analysis was applied.

Univariate analyses were initially performed to identify variables associated with adverse outcomes (RRT or mortality). Variables with *p* < 0.05 were further evaluated using receiver operating characteristic (ROC) analysis to determine optimal cut-off values based on Youden’s index. Variables meeting both statistical and clinical significance criteria were entered into a multivariate logistic regression model using forward stepwise (Wald) selection, with entry and removal thresholds of *p* < 0.05 and *p* < 0.10, respectively.

Regression coefficients were scaled relative to the smallest significant coefficient and rounded to the nearest 0.5 to construct a clinically interpretable scoring system. Non-significant predictors were assigned a score of zero.

The final scoring model’s performance was assessed using ROC curve analysis. Sensitivity, specificity, positive predictive value (PPV), negative predictive value (NPV), and F1 score were calculated for the optimal cutoff. Model calibration was evaluated using a calibration plot, which demonstrated reasonable agreement between predicted probabilities and observed event rates across most probability bins.

To reduce the risk of overfitting and ensure the robustness of the model, five-fold cross-validation was performed by randomly dividing the dataset into five equal parts, and a bootstrap resampling procedure (1000 iterations) was conducted to further evaluate model stability and estimate optimism in performance metrics.

All statistical tests were two-tailed, with significance set at *p* < 0.05. Analyses were conducted using SPSS version 27.0 (IBM Corp., Armonk, NY, USA), MedCalc version 20.1.4 (MedCalc Software Ltd., Ostend, Belgium), RStudio version 2024.09.0 (RStudio PBC, Boston, MA, USA), and MATLAB version 9.11 (R2021b) (MathWorks Inc., Natick, MA, USA).

## Results

**Fig. 1:**
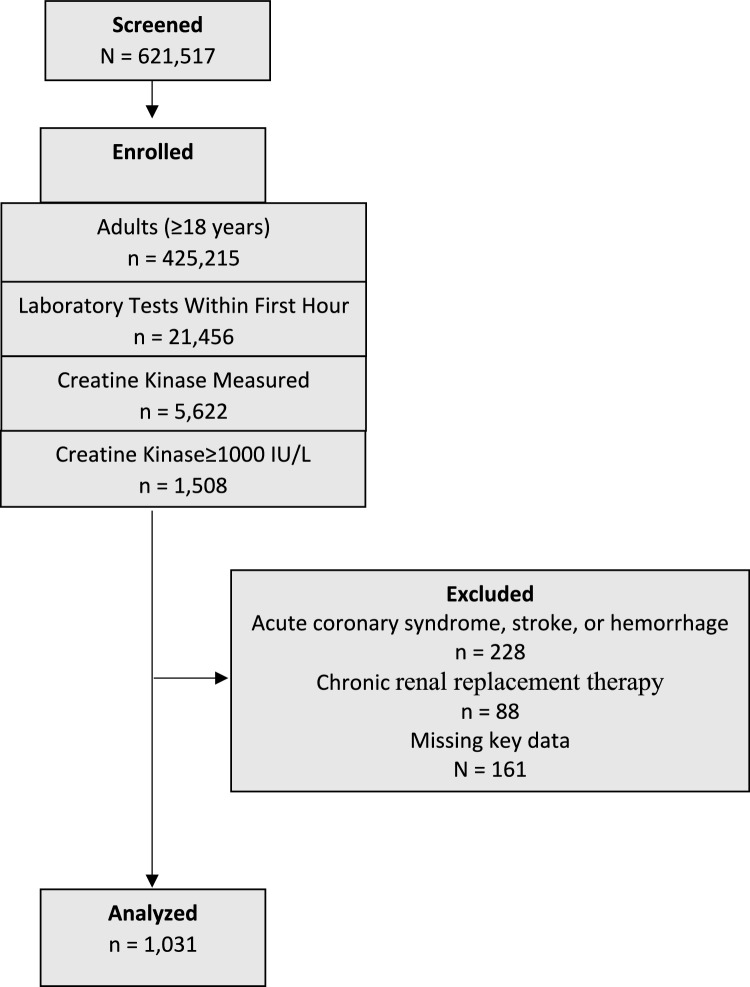
Flow diagram depicting patient selection for inclusion in the final analysis

Out of 1508 ED patients with serum creatine kinase (CK) levels > 1000 U/L, 1031 met eligibility criteria and were included in the final analysis. The patient selection process is summarized in Fig. 1. The mean age was 49.0 ± 21.8 years (range: 18–99), and 75.9% were male. The most common etiological categories were Group 2 (39.4%) and Group 5 (33.2%).

Composite adverse outcomes, defined as either RRT or all-cause mortality within 90 days, were observed in 109 patients (10.6%). Of the full cohort, 56 patients (5.5%) underwent RRT, while 84 patients (8.1%) died within the follow-up period. Among those who died, the median survival time was 4 days (range: 1–78 days). Patients with adverse outcomes were significantly older (median age 72 vs. 44 years, *p* < 0.001). The median CK level was 2627 U/L (range: 1000–54,986 U/L), and there was no significant difference in CK levels between outcome groups (*p* = 0.666). Hospitalization occurred in 53.1% of patients (*n* = 545), and 14.4% (*n* = 148) required ICU admission. The median LOS for hospitalized patients was 7 days (range: 1–144), and for ICU patients, 6 days (range: 1–144). Descriptive characteristics stratified by outcome status are detailed in Table 1.

**Table 1:** Demographic, clinical, and laboratory characteristics of study patients

Variables	Good outcomes (*n* = 922)	Adverse outcomes (*n* = 109)	*p*-value
Demographic dat			
Age (years)	44 (18–98)	72 (18–99)	** < 0.001**
Female/male	213/709	35/74	**0.037**
Etiological factor groups, *n* (%)			
Group 1	184^a^ (20)	47^b^ (43.1)	** < 0.001**
Group 2	373^a^ (40.5)	34^a^ (31.2)	
Group 3	15^a^ (1.6)	5^b^ (4.6)	
Group 4	25^a^ (2.7)	5^a^ (4.6)	
Group 5	325^a^ (35.2)	18^b^ (16.5)	
Laboratory results			
White blood cell count (× 10^3^/μL)	10.89 (0.10–63.79)	11.58 (0.27–46.44)	0.244
Hemoglobin (g/dL)	13.40 (4.20–18.40)	12 (3–18.5)	** < 0.001**
Platelet count (× 10^3^/μL)	225.5 (4–654)	183 (12–614)	** < 0.001**
MCHC (g/dL)	33.3 (25.8–39.9)	32.3 (24.8–37.5)	** < 0.001**
MPV (fl)	10.1 (7.8–14)	10.45 (8.5–14.5)	** < 0.001**
CK (U/L)	1549 (1000–54,986)	1538 (1000–16,636)	0.666
Glucose (mg/dL)	115 (25–580)	134 (30–1068)	** < 0.001**
Urea (mg/dL)	33 (3–408)	98.5 (18–559)	** < 0.001**
Creatinine (mg/dL)	0.94 (0.24–6.86)	2.19 (0.41–19.24)	** < 0.001**
GFR (mL/min)	90.55 (13.3–172.8)	24.0 (2.0–112.6)	** < 0.001**
Sodium (mEq/L)	137 (103–176)	137 (104–167)	0.877
Potassium (mEq/L)	4.2 (1.4–8.5)	4.2 (1.9–8.9)	0.224
Chloride (mEq/L)	100 (63–141)	100 (59–140)	0.355
Calcium (mg/dL)	9.0 (2.7–11.6)	8.0 (4.1–18.6)	** < 0.001**
ALP (U/L)	74 (20–719)	91 (25–1329)	** < 0.001**
Troponin T (ng/L)	9 (3–283)	91 (5–753)	** < 0.001**
D-dimer (ng/mL)	869 (10–9864)	3690 (324–9684)	** < 0.001**
pH	7.38 (6.80–7.69)	7.30 (6.80–7.52)	** < 0.001**
HCO_3_ (mmol/L)	24.2 (3–38.2)	17.3 (3.4–37.8)	** < 0.001**
BEecf (mmol/L)	− 0.1 (− 26.5–15)	− 7.45 (− 2.47 to 14.5)	** < 0.001**
Lactat (mmol/L)	1.6 (0.3–15)	2.2 (0.2–15)	**0.002**
Secondary outcomes			
Hospital LOS, d	0 (0–143)	6 (1–144)	** < 0.001**
ICU admission *n* (%)	101 (11)	80 (73.4)	** < 0.001**
ICU LOS, d	6 (1–123)	5 (1–144)	0.174

Data are expressed as median (minimum.-maximum.) frequency (percentage of the group subjects) for categorical variables unless otherwise indicated. The same letters in the same row indicates similarity between column proportions, different letters in the same row indicates statistical difference between column proportions

*MCHC* mean corpuscular hemoglobin concentration, *MPV* mean platelet volüme, *CK* creatinine Kinase, *GFR* glomerular riltration rate (mL/min/1.73 m^2^), *ALP* alkaline phosphatase, *HCO*_*3*_ bicarbonate, *BEecf* base excess of extracellular fluid, *LOS* length of stay, *ICU* intensive care unit

To develop the scoring system, univariate analyses were performed to identify variables significantly associated with adverse outcomes. Variables with *p* < 0.05 were considered candidate predictors. Subsequently, ROC curve analysis was employed to establish optimal cut-off values for the identified candidate predictors, with results summarized in Table 2 The variables that satisfied both statistical significance in univariate analysis and clinical relevance were as follows: age ≥ 50 years, platelet count ≤ 170 × 10^3^/μL, mean corpuscular hemoglobin concentration (MCHC) ≤ 32.8 g/dL, serum calcium ≤ 8.5 mg/dL, alkaline phosphatase (ALP) ≥ 115 U/L, base excess in the extracellular fluid (BEecf) ≤  − 6 mmol/L, and etiological categories corresponding to sepsis and toxic/metabolic causes (see Table 3).

**Table 2:** Optimal cut-off values and performance metrics for predictors of adverse outcomes

Variables	Cut-off	AUC (95% CI)	SE_AUC_	Sensitivity (%)	Specificity (%)	*p*-value
Age (years)	≥ 50	0.758 (0.731–0.784)	0.023	80.7	59.3	** < 0.001**
Hemoglobin (g/dL)	≤ 12.5	0.666 (0.636–0.694)	0.029	58.7	64.5	** < 0.001**
Platelet (× 10^3^/μL)	≤ 170	0.633 (0.602–0.662)	0.032	47.7	78.5	** < 0.001**
MCHC (g/dL)	≤ 32.8	0.680 (0.651–0.709)	0.028	66.6	64.1	** < 0.001**
MPV (fl)	≥ 10	0.645 (0.614–0.675)	0.028	73.4	47.1	** < 0.001**
Glucose (mg/dL)	≥ 122	0.596 (0.566–0.627)	0.033	64.2	59.1	**0.004**
Creatinine (mg/dL)	≥ 1.5	0.852 (0.829–0.873)	0.021	69.7	85.7	** < 0.001**
GFR (mL/min)	≤ 50	0.869 (0.846–0.889)	0.019	79.4	82.2	** < 0.001**
Calcium (mg/dL)	≤ 8,5	0.748 (0.721–0.775)	0.030	66.9	74.9	** < 0.001**
ALP (U/L)	≥ 115	0.644 (0.614–0.674)	0.032	41.6	87.6	** < 0.001**
Urea (mg/dL)	≥ 60	0.825 (0.801–0.848)	0.023	70.6	83.4	** < 0.001**
Troponin T (ng/L)	≥ 30	0.873 (0.843–0.899)	0.018	80.4	73.6	** < 0.001**
d-dimer (ng/mL)	≥ 1100	0.753 (0.689–0.811)	0.036	83.3	60.1	** < 0.001**
pH	≤ 7.30	0.658 (0.620–0.694)	0.034	50.9	79.2	** < 0.001**
BEecf (mmol/L)	≤ −6	0.719 (0.676–0.759)	0.035	57.1	81.6	** < 0.001**
HCO_3_ (mmol/L)	≤ 18	0.709 (0.672–0.744)	0.033	53.0	83.1	** < 0.001**
Lactat (mmol/L)	≥ 2	0.606 (0.561–0.650)	0.035	54.1	63.1	**0.002**

*MCHC* mean corpuscular hemoglobin concentration, *MPV* mean platelet volüme, *GFR* glomerular filtration rate (mL/min/1.73 m^2^), *ALP* alkaline phosphatase, *HCO3* bicarbonate, *BEecf* base excess of extracellular fluid, *AUC* area under the curve, *CI* confidence interval, *SE*_*AUC*_ standard error of the area under the curve

**Table 3:** Multivariate logistic regression analysis results for predictors of adverse outcomes

Predictors	Multivariate LR			β/minimum β		Allocated point
	β	OR (95% CI)	*p*-value			
Age, years (Reference: < 50)						
Age ≥ 50	0.843	2.323 (1.143–4.721)	**0.020**	0.843/0.584		**1.5**
Platelet, × 10^3^/μL (Reference: > 170)						
Platelet ≤ 170	0.694	2.001 (1.122–3.567)	**0.019**	0.694/0.584		**1**
MCHC, g/dL (Reference: > 32.8)						
MCHC ≤ 32.8	0.584	1.793 (1.004–3.202)	**0.048**	0.584/0.584		**1**
Calcium, mg/dL (Reference: > 8.5)						
Calcium ≤ 8.5	0.678	1.971 (1.095–3.548)	**0.024**	0.678/0.584		**1**
ALP, U/L (Reference: < 115)						
ALP ≥ 115	0.884	2.420 (1.303–4.497)	**0.005**	0.884/0.584		**1.5**
BEecf (mol/L (Reference: > − 6)						
BEecf ≤ − 6	1.496	4.463 (2.458–8.103)	** < 0.001**	1.496/0.584		**2.5**
Etiological factor groups (Reference: Group 5)			**0.014**			
Group 1	0.925	2.522 (1.167–5.450)	**0.019**	0.925/0.584		**1.5**
Group 2	− 0.095	0.910 (0.394–2.101)	0.824	0/0.584		**0**
Group 3	1.440	4.220 (1.117–15.942)	**0.034**	1.440/0.584		**2.5**
Group 4	0.717	2.049 (0.372–11.291)	0.410	0/0.584		**0**

Forward Wald method was used as variable selection to multivariate LR model

*MCHC* mean corpuscular hemoglobin concentration, *ALP* alkaline phosphatase, *BEecf* base excess of extracellular fluid, *OR* odds ratio, *CI* confidence interval, *Multivariate LR* multivariate linear regression

Seven variables remained independently associated with the outcome and were used to construct the final scoring system referred to as the Mugla Score. Points were assigned to each variable based on their standardized regression coefficients, resulting in a score ranging from 0 to 12.5. The scoring structure is presented in Table 4.

**Table 4:** Mugla Score for predicting adverse outcomes

Variables	Allocated point^a^
Age ≥ 50 years	**1.5**
Platelet ≤ 170 × 10^3^/μL	**1**
MCHC ≤ 32.8 g/dL	**1**
Calcium ≤ 8.5 mg/dL	**1**
ALP ≥ 115 U/L	**1.5**
BEecf ≤ − 6 mmol/L	**2.5**
Etiological factor groups^b^	
Group 1	**1.5**
Group 3	**2.5**

*ALP* Alkaline phosphatase, *MCHC* Mean Corpuscular Hemoglobin Concentration, *BEecf* base excess of extracellular fluid

^a^A total score individual patient score is obtained by summing the points for each variables

^b^Group 1, Alcohol consumption, endocrine disorders, metabolic disturbances, myopathies and infections; Group 3, Hyperosmolar and dehydration conditions

**Fig. 2:**
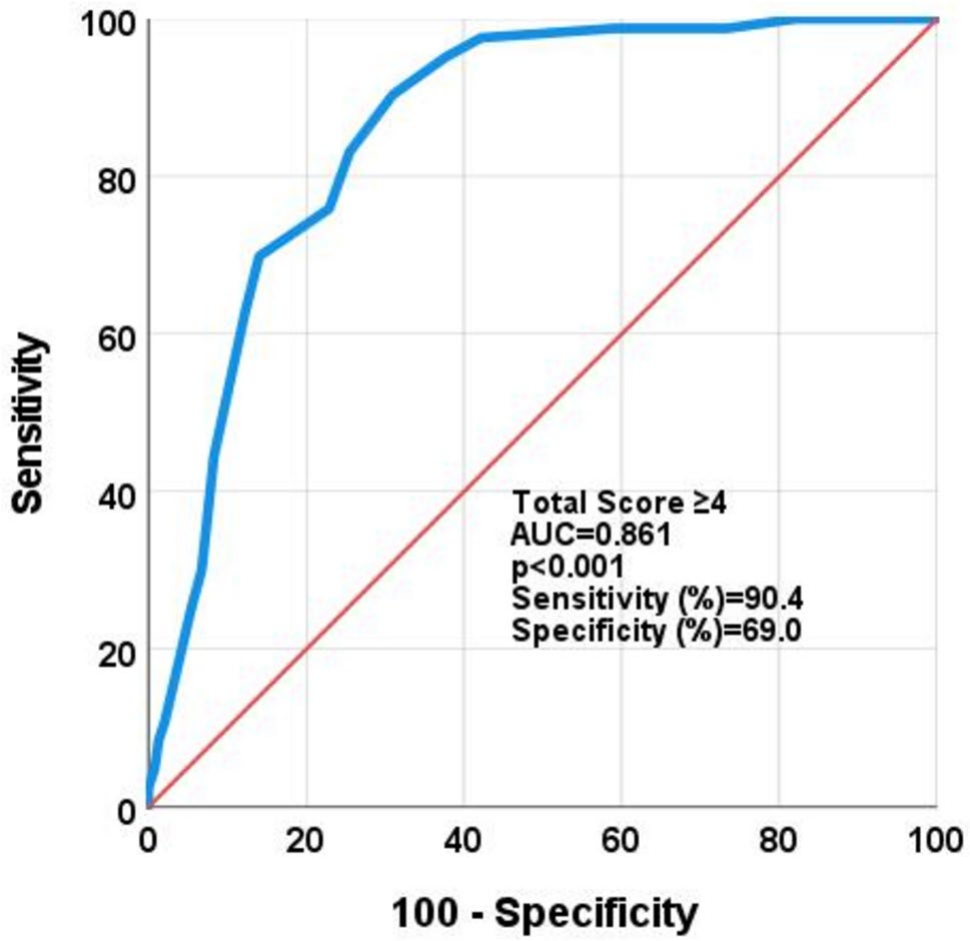
Area under the roc curve for the Mugla Score in predicting 3-month adverse outcomes

The Mugla Score demonstrated strong discriminatory ability with an AUC of 0.861 (95% CI: 0.824–0.898; *p* < 0.001). At the optimal cut-off of ≥ 4 points, the model achieved a sensitivity of 75.0%, specificity of 75.3%, PPV of 39%, NPV of 97%, and an F1 score of 0.541. Figure 2 illustrates the ROC curve for the final model.

**Fig. 3:**
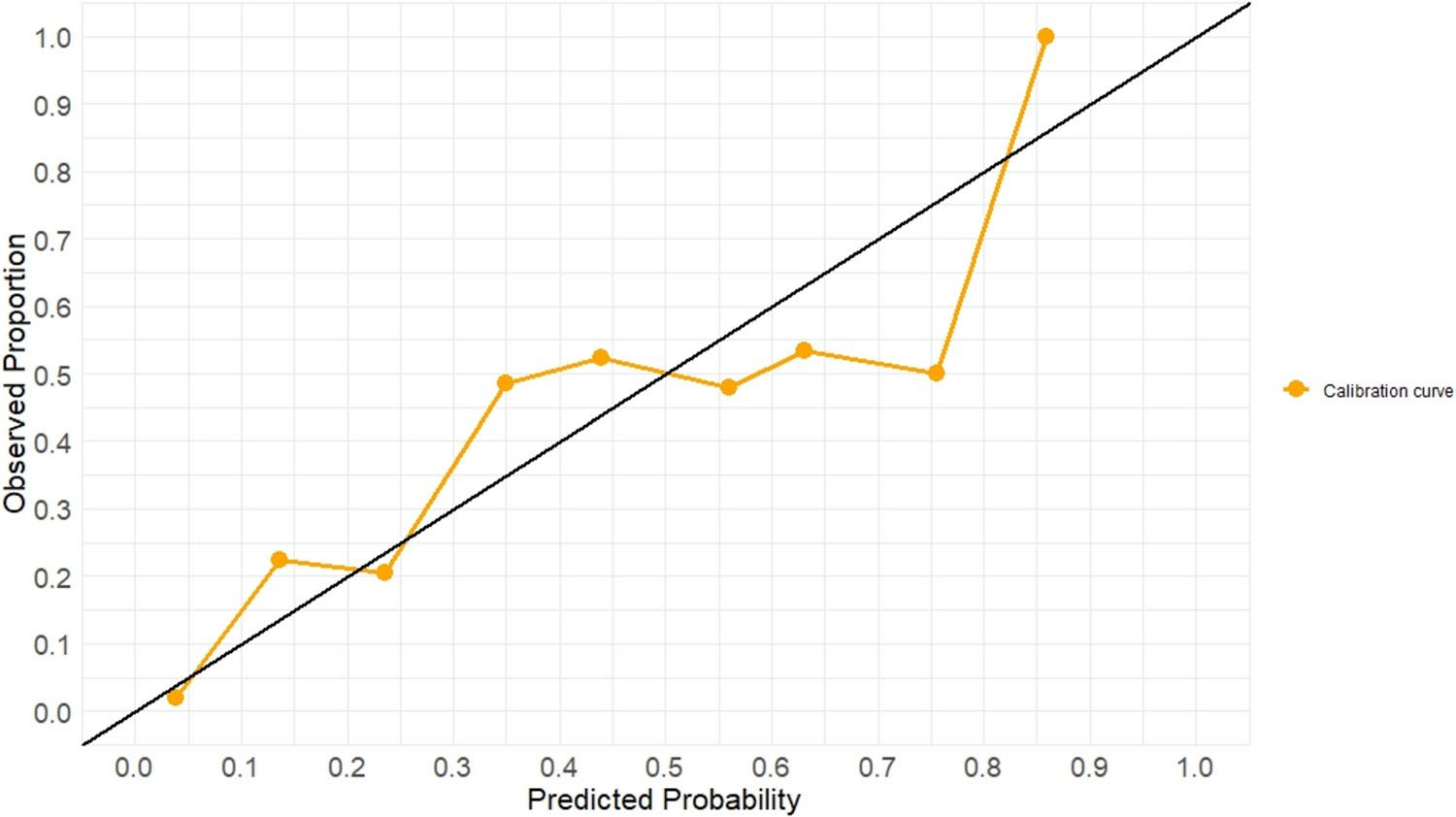
Calibration curve for predicted probabilities against observed outcomes

Calibration analysis showed that the predicted probabilities were in good agreement with observed event rates across most bins. The calibration curve demonstrated particularly accurate estimation in the mid-range risk probabilities (0.3–0.7), with a tendency to slightly overestimate risk at higher predicted probabilities (> 0.8). The visual calibration plot is presented in Fig. 3.

Internal validation using fivefold cross-validation yielded an AUC of 0.833 (95% CI: 0.796–0.866), with a sensitivity of 92.8% and specificity of 64.8%. The PPV and NPV in cross-validation were 36.3% and 97.7%, respectively. Furthermore, 1000-iteration bootstrap analysis was performed to test model robustness. The average AUC was 0.815, with a 95% confidence interval of 0.753–0.877, indicating strong generalizability and low risk of overfitting.

## Discussion

This study offers critical insights into the underexamined subset of rhabdomyolysis patients presenting to the ED, highlighting the urgent need for risk stratification tools that are both clinically relevant and operationally feasible. The Mugla Score was developed in response to this need, using readily accessible clinical, biochemical, and etiological data obtained during the first hour of ED care. It aims to assist frontline providers in identifying individuals at elevated risk for adverse outcomes—specifically, the need for RRT or three-month all-cause mortality—thereby supporting timely intervention and informed triage.

Several of the parameters incorporated into the model—platelet count, MCHC, calcium, ALP, and BEecf—reflect pathophysiological mechanisms relevant to rhabdomyolysis severity. Thrombocytopenia and low MCHC, although not traditionally emphasized in prognostic modeling, may represent early hematological markers of oxidative stress and systemic inflammation [7–9]. Hypocalcemia and elevated ALP similarly reflect dysregulated mineral metabolism and hepatic or musculoskeletal stress responses [10–14]. The prognostic significance of BEecf, a surrogate marker for metabolic acidosis, is consistent with prior evidence linking acid–base imbalance to impaired cellular function, tissue hypoperfusion, and adverse outcomes in critical illness [15, 16].

The etiological spectrum observed in this cohort challenges prior assumptions regarding the predominance of trauma as a high-risk contributor to rhabdomyolysis-related complications [4, 17]. Instead, non-traumatic factors—particularly dehydration, systemic infections, endocrinopathies, and COVID-19-associated myositis—were more strongly associated with adverse events [18–22]. This finding reinforces the importance of integrating etiological classification into prognostic models and affirms the need for context-sensitive risk assessment in heterogeneous clinical populations.

Recent efforts to model adverse outcomes in rhabdomyolysis have reflected two dominant paradigms: data-driven prognostication using complex algorithms, and traditional regression-based approaches emphasizing clinical transparency. In this context, Liu et al. and McMahon et al. represent influential contributions from ICU-centered perspectives—one favoring machine learning, the other classical scoring. While both models demonstrated strong predictive performance in critically ill populations, their design priorities inherently limit generalizability to early-phase ED care [4, 5]. In contrast, the Mugla Score was constructed to balance statistical rigor with bedside practicality, using only point-of-care data available during the first hour of triage. This emphasis on immediacy enables the score to function not merely as a prognostic classifier but as a real-time decision-support tool. Moreover, by incorporating etiological subtypes alongside biochemical and hematologic markers, the model reflects the clinical heterogeneity of rhabdomyolysis in unselected ED cohorts. These methodological distinctions underscore the need for stratification tools tailored to specific phases of care—ranging from high-complexity ICU models to ED-integrated frameworks optimized for rapid deployment.

The Mugla Score was developed to align with the operational tempo of EDs, emphasizing rapid access to decision-support without reliance on delayed or non-routine parameters. Its application requires no advanced computational infrastructure, and its components are derived entirely from laboratory and clinical data routinely collected within the first hour of triage. This structure allows the score to function both as a clinically interpretable stratification framework and as a pragmatic tool for early risk communication. From an implementation standpoint, efforts are in progress to develop a mobile-responsive digital calculator, enabling point-of-care use of the Mugla Score. Such a tool may facilitate real-time decision-making in EDs by offering rapid risk estimation, supporting disposition planning, and informing nephrology consultation and resource allocation. Embedding the score into clinical decision support systems may also enhance adherence and scalability in high-throughput acute care settings.

### Limitations

This study has several limitations that merit careful consideration. First, the model was developed using data from a retrospective, single-center cohort, which may introduce selection bias and limit the generalizability of findings beyond the institutional setting in which it was constructed. Although internal validity was enhanced through five-fold cross-validation and bootstrap resampling, external validation has not yet been performed. Prospective, multicenter studies will be necessary to evaluate the model’s performance in broader clinical contexts. Furthermore, although the proportion of missing data was low and statistical analysis supported the assumption of MCAR, the use of complete case analysis may have reduced statistical power by decreasing the effective sample size.

Second, while the model was intentionally constructed using variables routinely available within the first hour of ED evaluation, certain clinically relevant predictors—such as serum phosphate, urine output, and comorbidity indices—were not included due to inconsistent documentation or unavailability in the acute phase. This absence not only restricted the feature set used in model development but also precluded head-to-head comparison with existing tools such as the McMahon and Liu Scores, which require these omitted parameters. As a result, direct benchmarking across models was not feasible, and performance comparisons must be interpreted with caution.

Third, although all laboratory values used in the model were collected at the ED presentation, the time interval between symptom onset and hospital arrival could not be reliably standardized across patients. This limitation prevents precise alignment between the clinical trajectory of muscle injury and the biochemical profile captured at triage, potentially introducing temporal misclassification and affecting model calibration.

Fourth, while predictor selection followed methodologic rigor and cut-off points were statistically optimized via ROC analysis, the categorization of continuous variables may reduce flexibility in individualized risk estimation. Stepwise regression, though potentially prone to model instability and biased selection, was chosen for its clinical interpretability and compatibility with point-based tools. However, future studies may consider penalized approaches such as LASSO to improve stability, particularly in high-dimensional or sparse data contexts. Furthermore, although the model integrates variables with plausible biological relevance, residual confounding due to unmeasured or unrecorded clinical factors cannot be excluded.

## Conclusion

The Mugla Score provides a clinically interpretable, ED–oriented tool for early risk stratification in patients with rhabdomyolysis. Developed using routinely available laboratory and clinical data, and incorporating etiological diversity, the model demonstrated strong internal validity and practical relevance at the bedside. Its simplicity, rapid applicability, and reliance on first-hour triage data enhance its utility in time-sensitive, resource-constrained environments. Although external validation is needed to confirm generalizability, the Mugla Score represents a promising step toward real-time, risk-adapted decision-making in acute care. It may inform early nephrology consultation, ICU triage, and targeted resuscitation strategies in this high-risk population. 

## Supplementary Information

Below is the link to the electronic supplementary material.

Supplementary file1 (DOCX 22 KB)
