# Supplementary material for: Development of the Mugla Score: an association-based tool for risk stratification in emergency department patients with rhabdomyolysis
Source: Intern Emerg Med. 2025 Jun 11;21(3):1087–96. doi: 10.1007/s11739-025-04009-y (PMC13144174; doi:10.1007/s11739-025-04009-y)
Supplement: Supplementary file 1 — Supplementary file1 (DOCX 22 KB) [file 11739_2025_4009_MOESM1_ESM.docx]

**Appendix A:** Includes structured definitions and representative examples for all five etiological categories.

| **Group** | **Etiological Category** | **Definition** | **Representative Conditions** |
| --- | --- | --- | --- |
| **Group 1** | Metabolic, Infectious, and Myopathic Causes | Includes endocrine disorders, metabolic and mitochondrial abnormalities, infectious agents, and primary myopathies. | COVID-19, dermatomyositis, glycogen storage diseases, HIV, hyperthyroidism, hypothyroidism, influenza, mitochondrial disorders, polymyositis |
| **Group 2** | Trauma- and Exertion-Related Causes | Involves direct trauma (including surgical procedures), seizure-related activity, and intense physical exertion. | Crush injuries, electrical burns, high-intensity interval training (e.g., spinning), marathon running, motor vehicle collisions, prolonged seizures, status epilepticus |
| **Group 3** | Dehydration- and Hyperthermia-Associated States | Includes fluid depletion and thermal dysregulation in environmental or iatrogenic contexts. | Heat stroke, malignant hyperthermia, neuroleptic malignant syndrome, prolonged exposure to high ambient temperatures, severe dehydration |
| **Group 4** | Drug-, Toxin-, and Venom-Related Causes | Encompasses prescribed and recreational drugs, envenomation, and environmental or ingested toxins. | Amphetamines, cocaine, haloperidol, heavy metals, scorpion venom, snake venom, SSRIs, statins, synthetic cannabinoids, toxic mushrooms, foodborne toxins |
| **Group 5** | Miscellaneous or Unclassified Causes | Includes idiopathic presentations or cases not attributable to established categories. | Haff disease, idiopathic rhabdomyolysis, multifactorial etiologies involving minor interacting triggers |

Abbreviations; COVID-19, Coronavirus Disease 2019; HIV, Human Immunodeficiency Virus; SSRIs, Selective Serotonin Reuptake Inhibitors
